# Are Risk Factors Associated with Outcomes in Pancreatic Cancer?

**DOI:** 10.1371/journal.pone.0041984

**Published:** 2012-07-24

**Authors:** De-shen Wang, Zhi-qiang Wang, Le Zhang, Miao-zhen Qiu, Hui-yan Luo, Chao Ren, Dong-sheng Zhang, Feng-hua Wang, Yu-hong Li, Rui-hua Xu

**Affiliations:** State Key Laboratory of Oncology in South China, Department of Medical Oncology, Sun Yat-sen University Cancer Center, Guangzhou, China; Drexel University College of Medicine, United States of America

## Abstract

**Background:**

The development of pancreatic cancer is a process in which genes interact with environmental factors. We performed this study to determine the effects of the ABO blood group, obesity, diabetes mellitus, metabolic syndrome (MetS), smoking, alcohol consumption and hepatitis B viral (HBV) infection on patient survival.

**Methods:**

A total of 488 patients with pancreatic cancer were evaluated.

**Result:**

Patients who presented as chronic carriers of HBV infection were younger at disease onset (*p = *0.001) and more predominantly male (*p = *0.020) than those never exposed to HBV. Patients with MetS had later disease staging (*p = *0.000) and a lower degree of pathological differentiation (*p = *0.008) than those without MetS. In a univariate analysis, the ABO blood group, smoking and alcohol consumption were not associated with overall survival. HBsAg–positivity and elevated fasting plasma glucose were significantly associated with unfavorable survival though not in the multivariate analysis. The presence of MetS (HR: 1.541, 95% CI: 1.095–2.169, *p = *0.013), age ≥65, an elevated CA19–9 baseline level, TNM staging, the type of surgery, the degree of differentiation and chemotherapy were independently associated with overall survival.

**Conclusion:**

We report, for the first time, that patients with chronic HBV infection may represent a special subtype of pancreatic cancer, who have a younger age of disease onset and male dominancy. Patients with MetS had later disease staging and a poorer histological grade. Patients with MetS demonstrated significantly poorer survival.

## Introduction

Pancreatic cancer is the fifth most common cancer and the fourth leading cause of cancer-related mortality for men and women in Western societies [1], with nearly 227000 deaths per year [2]. At present, surgical resection remains the only approach to curing this disease. However, less than 20% of patients present with early disease onset. The prognosis remains poor, and conventional treatments have little impact on the disease process. Therefore, it is important to understand the intrinsic properties of cancer cells that influence the progression of the tumor and to identify more accurate prognostic factors for more effective therapies.

It has been increasingly recognized that the development of cancer is a process in which genes interact with environmental factors. Several risk factors related to pancreatic cancer have been previously explored, including genetics [3], somatostatin receptor 5 gene polymorphisms [4], alcohol intake [5], cigarette smoking [6], diabetes mellitus [7], obesity [8], metabolic syndrome (MetS) [9], [10], [11], [12], chronic pancreatitis [13], first-degree relatives with pancreatic cancer [14], exposure to poultry oncogenic viruses [15], and Helicobacter pylori infection [16]. In recent years, several studies have found an association between ABO blood group antigens and hepatitis B viral (HBV) infection and the risk of pancreatic cancer [17], [18], [19], [20], [21], [22], [23]. However, risk factors may also be seen as candidate prognostic factors. An in vitro study found that hyperglycemia and diabetes may stimulate pancreatic cancer cell proliferation and confer resistance to gemcitabine [24]. Nevertheless, the role of diabetes mellitus in the outcomes of patients with pancreatic cancer has been largely unclear [25], [26], [27], [28]. Obesity is a risk factor and a prognostic factor for patients with pancreatic cancer [29], [30]. Adiposity in obese patients has been shown to be strongly associated with insulin resistance and lipid metabolism disorders [31]. This group of risk factors, which commonly appear together, has been defined as metabolic syndrome (MetS) [32]. It is increasingly recognized that metabolic tissue can activate the insulin growth factor signaling pathway [33] and proinflammatory mediators, including macrophages, T cells and tumor necrosis factor-alpha [34], [35], which, in turn, create a favorable microenvironment for tumor development and progression. MetS has been identified as an independent risk factor for pancreatic cancer in some population-based studies [9], [10], [11], [12]. However, evidence for the association between MetS and cancer-related survival in patients with pancreatic cancer has been sparse. Moreover, two studies investigating the prognostic value of the ABO blood type and pancreatic cancer have shown inconsistent results [36], [37]. In particular, the impact of HBV infection on the overall survival of patients with pancreatic cancer has not been well evaluated.

Therefore, we performed this study including patient clinicopathological characteristics and survival dates to determine the prognostic value of several risk factors related to pancreatic cancer.

## Patients and Methods

### Ethics Statement

Clinical and laboratory examinations were performed after obtaining informed written consent from all patients and approval from the independent Institute Research Ethics Committee at the Cancer Center of Sun Yat-sen University.

### Study Population

Between January 1, 1998, and December 30, 2010, 645 patients with histologically confirmed pancreatic adenocarcinoma treated at the Sun Yat-sen University Cancer Center in Guangzhou, China, were retrospectively analyzed. Patient follow-up was completed until October 1, 2011. At the end of follow-up, survival data were unavailable for 157 patients due to incorrect contact information. A total of 488 patients with complete survival dates were included in this study. A 3-dimensional, contrast-enhanced computed tomography (CT) scan or magnetic resonance imaging (MRI) scan was used for clinical staging. In patients who were considered unresectable, pathological assessment was based on a cytological diagnosis through fine-needle aspiration. A biopsy specimen was used for pathological diagnosis in patients who had undergone exploration only. In patients who had undergone surgical resection, the pathological diagnosis depended on the resected specimen.

Serum samples were collected to test the ABO blood groups and the presence of HBV infection. The ABO blood types (i.e., A, B, AB and O) were tested using mouse-derived monoclonal antibodies (Ortho Bioclones anti-A, B, and O, Ortho Diagnostic Systems Inc., Raritan, NJ, USA). An enzyme-linked immunosorbent assay was used to detect hepatitis B surface antigen (HBsAg), hepatitis B surface antibody (anti-HBs), hepatitis B e antigen (HBeAg), hepatitis B e antibody (anti-HBe), and hepatitis B core antibody (anti-HBc) (Kehua Bio-Engineering Co., Ltd., Shanghai, China). Data on patients’ age, sex, baseline blood pressure, height, weight, fasting plasma glucose, triglyceride and HDL levels, cigarette smoking, alcohol intake, personal history of diabetes mellitus and hypertension were collected by clinical staff.

### Definition of Metabolic Syndrome

Metabolic syndrome was defined as a clustering of three or more of the following five risk factors: (1) fasting plasma glucose ≥5.6 mmol/l (100 mg/dl) or currently taking medication for diabetes mellitus; (2) blood pressure ≥130/≥85 mmHg or currently taking medication for hypertension; (3) triglycerides ≥1.7 mmol/l (150 mg/dl); (4) HDL-cholesterol: for men: <1.03 mmol/l (40 mg/dl) and for women: <1.29 mmol/l (50 mg/dl); (5) obesity: for men: waist circumference >102 cm and for women: waist circumference >88 cm, which was suggested by the National Cholesterol Education Program Adult Treatment Panel (NCEP-ATP) III guidelines [32]. Because the waist-circumference measurements were not readily collected in our medical records, the body mass index (BMI) (i.e., ≥30) served as the proxy variable [38]. BMI was calculated as weight (kg) divided by height (m)^2^ and categorized into four groups (i.e., <18.5, underweight; 18.5 to 25, normal; ≥25 and <30, overweight; ≥30, obese).

### Statistical Analysis

Descriptive statistics of the clinicopathological data of patients with pancreatic cancer were calculated with mean, standard deviation (SD) and frequencies, depending on the type of data. To test whether the clinicopathological characteristics differed for those with HBV infection and MetS, Pearson’s χ^2^ test and Student’s t-test were used to compare the variables. The statistical analyses were performed using the SPSS statistical package (SPSS Inc., Chicago, IL, USA, version 16.0). Overall survival (OS) was calculated from the date of diagnosis to the date of patient death from cancer or the last date of follow-up. A Cox regression was used for univariate analysis. Variables that were significantly prognostic in the univariate analysis were selected for use in the final multivariable Cox proportional hazards regression analysis using the forward stepwise method. OS curves were compared using the two-sided log-rank test and Kaplan-Meier survival analyses. *P*<0.05 was defined as statistically significant.

## Results

The associations among clinicopathological characteristics, potential risk factors and OS of patients with pancreatic cancer are shown in Table 1. There were 332 (68.03%) males and 156 (31.97%) females. A total of 150 patients were older than 65 years (30.74%). Three-hundred patients (61.48%) experienced pretherapeutic weight loss ≥5 percent. Eighteen patients (3.69%) were categorized as obese. A total of 125 patients (25.61%) had a fasting plasma glucose levels ≥5.6 mmol/l (100 mg/dl), and 149 patients (30.53%) had triglyceride levels ≥1.7 mmol/l (150 mg/dl). Hypertension was present in 101 patients (20.70%). A total of 129 patients (26.43%) had low HDL levels. Seventy-three patients (14.96%) presented with MetS. HBsAg was positive in 64 patients (13.76%). Anti-HBc was positive in 199 patients (42.80%). A total of 166 patients (34.66%) were O blood type, and 313 patients (65.34%) were non-O blood type. HBsAg-positivity/anti-HBc-positivity was found in 64 patients (13.76%). Three-hundred forty-four patients (70.50%) had an elevated baseline CA199. The majority of tumors were located in the head of pancreas (321; 65.78%). A total of 91 (18.65%) patients had received macroscopically radical surgery. Three-hundred forty-five patients (70.70%) presented with celiac axis, superior mesenteric artery invasion or distant metastasis in the initial diagnosis. A total of 205 patients (42.01%) had received chemotherapy, and among them, 18 patients had also received adjuvant chemotherapy after resection.

**Table 1:** The association among clinicopathological characteristics, potential pancreatic cancer risk factors and overall survival in a univariate analysis.

		Overall Survival	
Factors	Number	Hazard ratio (95% confidence interval)	*P* value
**Age (<65/≥65)**	**338/150**	**1.394 (1.131–1.717)**	**0.002**
Gender (Male/Female)	332/156	0.920 (0.742–1.142)	0.451
**Pretherapeutic weight loss (Normal or loss <5 per cent/loss ≥5 per cent)**	**188/300**	**1.411 (1.148–1.734)**	**0.001**
ABO blood type (A+AB/O)	183/166	1.135 (0.896–1.437)	0.295
ABO blood type (B+AB/O)	155/166	1.184 (0.928–1.511)	0.173
ABO blood type (non-O/O)	313/166	1.150 (0.931–1.420)	0.194
**Hepatitis B Virus (HBV)**			
**HBV (HBsAg–negative/HBsAg–positive)**	**401/64**	**1.367 (1.024–1.826)**	**0.034**
HBV (Anti-HBs–negative/Anti-HBs–positive)	260/205	0.857 (0.696–1.055)	0.146
HBV (Anti-HBc–negative/Anti-HBc–positive)	266/199	1.027 (0.835–1.264)	0.799
HBV (HBsAg-negative and Anti-HBc–negative/HBsAg-positive and Anti-HBc–positive)	266/64	1.331 (0.987–1.794)	0.059
HBV (HBsAg-negative and Anti-HBc–negative/Anti-HBs–positive and Anti-HBc–positive )	266/123	0.942 (0.738–1.204)	0.635
Body mass index (BMI)			0.139*
≥18.5 and <25	340	1 (reference)	
<18.5	109	1.186 (0.932–1.508)	0.165
≥25.0 and <30	21	0.880 (0.523–1.481)	0.629
≥30	18	1.636 (0.984–2.718)	0.058
**Fasting plasma glucose**			**0.006** *
**<5.6 mmol/l (<100 mg/dl)**	**363**	1 (reference)	
**5.6–6.0 mmol/l (100–109 mg/dl)**	**30**	**1.161 (0.771–1.748)**	**0.476**
**6.1–6.9 mmol/l (110–125 mg/dl)**	**16**	**1.692 (0.986–2.903)**	**0.056**
**≥7.0 mmol/l (≥126 mg/dl)**	**79**	**1.535 (1.177–2.002)**	**0.002**
Blood pressure			0.288*
<130/85 mmHg	387	1 (reference)	
130/85–159/99 mmHg	89	1.212 (0.942–1.560)	0.135
160/100–179/109 mmHg	10	1.393 (0.690–2.814)	0.356
**≥**180/110****mmHg	2	2.040 (0.507–8.214)	0.316
Triglycerides			0.124*
0.56–1.7 mmol/l (50–150 mg/dl)	326	1 (reference)	
<0.56 mmol/l (<50 mg/dl)	13	1.357 (0.776–2.375)	0.284
≥1.7 mmol/l (≥150 mg/dl)	149	1.227 (0.990–1.521)	0.061
HDL-cholesterol			0.084*
Men: ≥1.03 mmol/l (40 mg/dl); Women: ≥1.29 mmol/l (50 mg/dl)	359	1 (reference)	
Men: <1.03 mmol/l (40 mg/dl); Women: <1.29 mmol/l (50 mg/dl)	129	1.216 (0.974–1.519)	0.084
**Number of Metabolic Syndrome (MetS) Components**			**0.000** *
0	224	1 (reference)	
1	100	0.876 (0.667–1.150)	0.341
2	91	0.938 (0.713–1.234)	0.646
** 3**	**60**	**1.910 (1.403–2.599)**	**0.000**
** 4**	**11**	**2.287 (1.238–4.224)**	**0.008**
** 5**	**2**	**8.834 (2.157–36.177)**	**0.002**
**Metabolic syndrome (MetS) (No/Yes)**			**0.000**
** No**	**415**	**1 (reference)**	
** Yes**	**73**	**2.101 (1.609–2.743)**	
Smoking status (Never/Current or past)	333/155	1.012 (0.819–1.250)	0.914
Alcohol drinking (Never/Current or past)	387/101	1.160 (0.913–1.474)	0.225
History of chronic pancreatitis (No/Yes)	486/2	1.345 (0.189–9.595)	0.767
First-degree relatives of pancreatic cancer (No/Yes)	484/4	0.574 (0.143–2.304)	0.433
A family history of other cancers (No/Yes)	428/60	0.925 (0.682–1.255)	0.619
**Baseline carcinoembryonic antigen 199 (CA19–9) (Normal/Elevated)**	**118/344**	**1.901 (1.474–2.451)**	**0.000**
Location of tumor (Head/Body/Tail/Diffuse)	321/74/56/37	1.105 (0.994–1.227)	0.063
**The 7^th^ tumor-node-metastasis (TNM) staging (AJCC) (Ia + Ib/IIa + IIb/III/IV)**	**13/130/134/211**	**1.819 (1.616–2.048)**	**0.000**
**Surgery (Macroscopically radical surgery/Bypass or stent only or exploration or none)**	**91/397**	**4.116 (2.985–5.677)**	**0.000**
**Degree of differentiation (Well differentiated/Moderate differentiated/Poorly differentiated or mucinous adenocarcinoma/Not documented)**	**45/117/185/141**	**1.643 (1.376–1.961)**	**0.000**
**Chemotherapy (No/Yes)**	**283/205**	**0.786 (0.643–0.962)**	**0.019**

* *p* values for trends.

**Figure 1:**
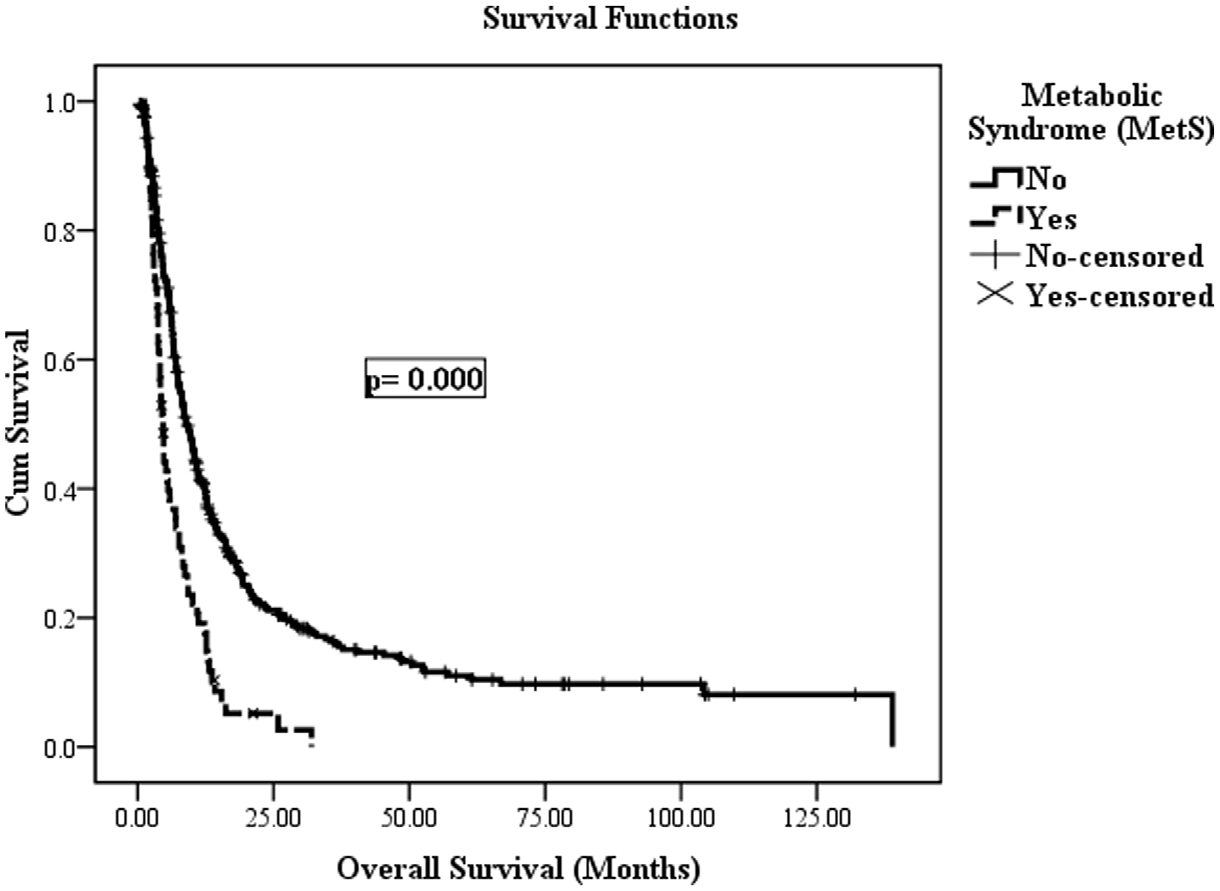
The overall survival comparing patients with metabolic syndrome (MetS) and those without in a univariate analysis.

In the univariate analysis, there was a graded increase in cancer-related mortality associated with a greater number of MetS components, and those with more than two components had a higher risk compared to patients with 0 components (*p* trend = 0.000). According to the univariate analysis, factors associated with OS included the presence of MetS (*p = *0.000) (Figure 1), HBsAg-positivity (*p = *0.034), age ≥65 (*p = *0.002), pretherapeutic weight loss ≥5 percent (*p = *0.001), elevated fasting plasma glucose (*p* trend = 0.006), elevated baseline CA19–9 levels (*p = *0.000), higher TNM staging (AJCC) (*p = *0.000), type of surgery (*p = *0.000), degree of differentiation (*p = *0.000) and chemotherapy (*p = *0.019). In addition, there was a trend toward chronic carriers of HBV infection (i.e., HBsAg-positive/anti-HBc-positive) having a negative effect on prognosis compared with patients who were never exposed to HBV (i.e., HBsAg-negative/anti-HBc-negative) (*p = *0.059). Patients with obesity, elevated levels of triglycerides and low levels of HDL were also shown have a likely shorter survival duration (*p = *0.058, *p = *0.061, and *p = *0.084, respectively). However, ABO blood type, gender, smoking, alcohol intake, history of chronic pancreatitis, family history of pancreatic and other cancers, and tumor location were not associated with overall survival (Table 1).

Although elevated fasting plasma glucose was a significant predictor of OS in the univariate analysis, it did not remain independent of OS in the multivariate analysis, which included fasting plasma glucose after adjustment for covariates except for other components of the metabolic syndrome (*p* trend = 0.188). When MetS was included in the multivariate model, it remained significant and independently associated with cancer mortality (HR: 1.541, 95% CI: 1.095–2.169, *p = *0.013). Otherwise, age ≥65 (*p = *0.032), elevated baseline CA19–9 levels (*p = *0.046), higher TNM staging (AJCC) (*p = *0.010), type of surgery (*p = *0.039), degree of differentiation (*p = *0.006) and chemotherapy (*p = *0.015) were also independently associated with OS. However, HBsAg-positivity was not an independent prognostic factor (*p = *0.711) (Table 2).

**Table 2:** A multivariate analysis of prognostic variables based on clinicopathological characteristics, potential risk factors and overall survival in patients with pancreatic cancer.

	Overall Survival	
Factors	Hazard ratio (95% confidence interval)	*P* value
**Age**		**0.032**
** <65**	**1 (reference)**	
** ≥65**	**1.370 (1.027–1.828)**	
Pretherapeutic weight loss		0.332
Normal or loss <5 per cent	1 (reference)	
Loss ≥5 per cent	1.146 (0.870–1.509)	
Hepatitis B Virus (HBV)		0.711
HBsAg–negative	1 (reference)	
HBsAg–positive	1.071 (0.745–1.540)	
Fasting plasma glucose *		0.188
<5.6 mmol/l (<100 mg/dl)	1 (reference)	
5.6–6.0 mmol/l (100–109 mg/dl)	1.034 (0.628–1.701)	0.896
6.1–6.9 mmol/l (110–125 mg/dl)	1.650 (0.833–3.265)	0.151
≥7.0 mmol/l (≥126 mg/dl)	1.371 (0.975–1.927)	0.070
**Metabolic syndrome (MetS)**		**0.013**
** No**	**1 (reference)**	
** Yes**	**1.541 (1.095–2.169)**	
**Baseline carcinoembryonic antigen 199 (CA19–9)**		**0.046**
** Normal**	**1 (reference)**	
** Elevated**	**1.399 (1.006–1.946)**	
**The 7^th^ tumor-node-metastasis (TNM) staging (AJCC)**		**0.010**
** Ia + Ib**	**1 (reference)**	
** IIa + IIb**	**1.929 (0.746–4.992)**	**0.175**
** III**	**3.239 (1.097–9.564)**	**0.033**
** IV**	**4.321 (1.452–12.858)**	**0.009**
**Surgery**		**0.039**
** Macroscopically radical surgery**	**1 (reference)**	
** Bypass or stent only or exploration or none**	**1.843 (1.033–3.290)**	
**Degree of differentiation**		**0.006**
** Well differentiated**	**1 (reference)**	
** Moderate differentiated**	**1.305 (0.833–2.045)**	**0.246**
** Poorly differentiated or mucinous adenocarcinoma**	**1.847 (1.195–2.854)**	**0.006**
**Chemotherapy**		**0.015**
** No**	**1 (reference)**	
** Yes**	**0.724 (0.558–0.939)**	

* Elevated fasting plasma glucose did not remain independent of overall survival in the multivariate analysis, which included fasting plasma glucose after adjustment for covariates except for other components of the metabolic syndrome (*p* trend = 0.188).

**Figure 2:**
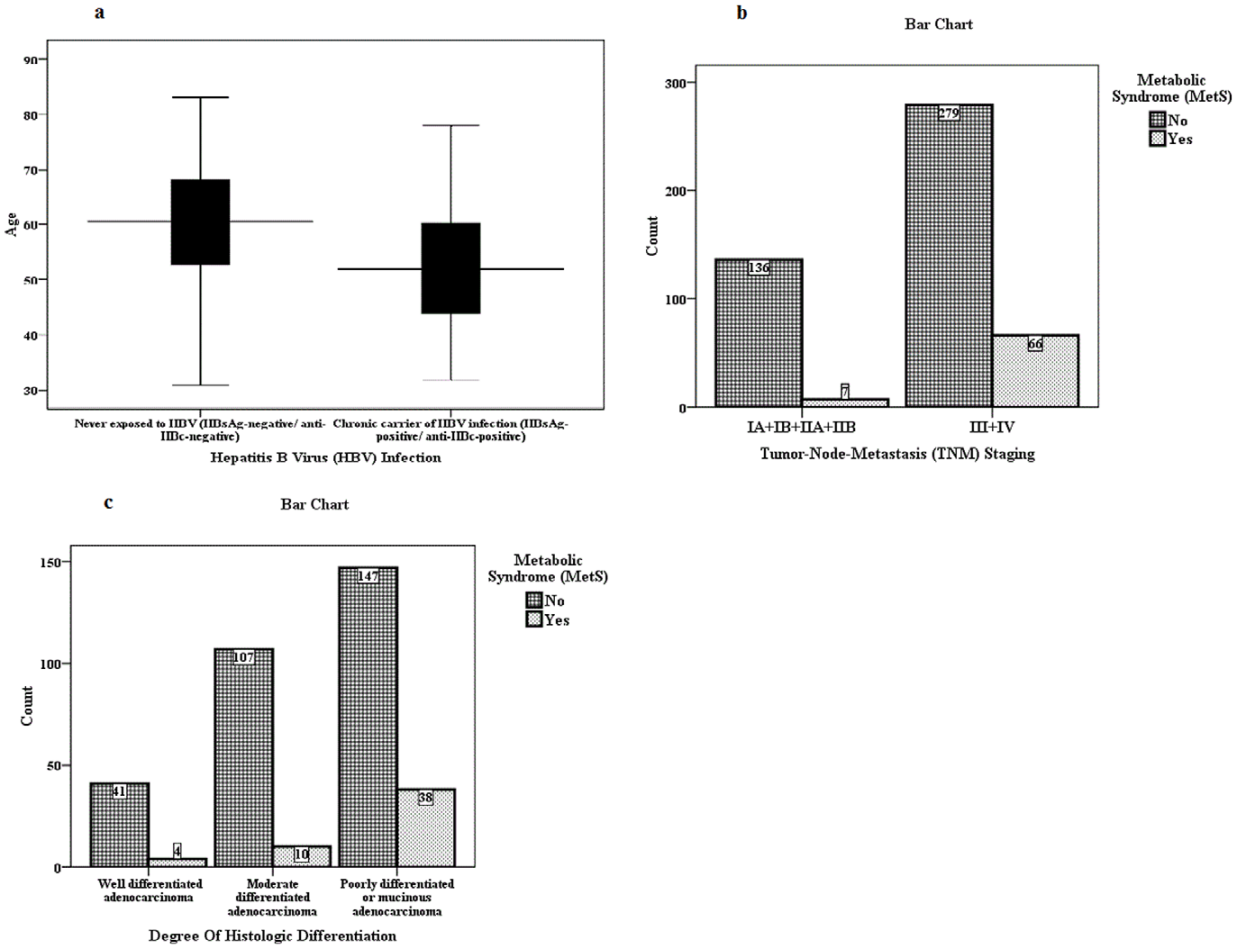
(A) The median age according to hepatitis B viral (HBV) infection status in patients with pancreatic cancer. The boxes represent values between the 25th and 75th percentiles, and the horizontal lines within the boxes indicate the median value. The median age (±standard deviation) of patients with HBsAg-positivity/anti-HBc-positivity was 52.00±11.155 years old, and for those with HBsAg-negativity/anti-HBc-negativity, it was 60.50±10.747 years old (*p = *0.001). (B) The association between the presence of metabolic syndrome (MetS) and tumor-node-metastasis (TNM) staging in patients with pancreatic cancer. A total of 66 (90.41%) patients presenting with MetS were stage III or IV compared with 279 (67.23%) patients who did not have MetS (*p = *0.000). (C) The association between the presence of metabolic syndrome (MetS) and the degree of histological differentiation in patients with pancreatic cancer. Dedifferentiated histology was more frequent in patients with than without MetS. A total of 38 (73.10%) patients presenting with MetS were poorly differentiated or had mucinous adenocarcinoma compared with 147 (49.80%) patients without MetS (*p = *0.008).

The association among the presence of MetS, HBV infection and the clinicopathological parameters of patients with pancreatic cancer are shown in Table 3. Patients who presented as chronic carriers of HBV infection (i.e., HBsAg-positive/anti-HBc–positive) were younger at disease onset and more predominantly male than those never exposed to HBV (i.e., HBsAg-negative/anti-HBc–negative). The median age (±SD) of patients with HBsAg-positivity/anti-HBc-positivity was 52.00±11.155 years old, and for those with HBsAg-negativity/anti-HBc-negativity, it was 60.50±10.747 years old (*p = *0.001) (Figure 2a). A total of 53 patients (82.81%) who were chronic carriers of HBV infection were male, and there were only 181 (68.05%) male patients who were never exposed to HBV (*p = *0.020). HBV infection was not significantly associated with the other clinicopathological characteristics of patients with pancreatic cancer. Patients who were older than 65 years old more frequently presented with MetS compared with patients who were younger than 65 years old (*p = *0.038). Sixty-six (90.41%) patients who presented with MetS were at stage III or IV compared with 279 (67.23%) patients who were not (*p = *0.000) (Figure 2b). Patients who presented with MetS had a poorer pathological differentiation grade than those without MetS (*p = *0.008) (Figure 2c) (Table 3).

**Table 3:** The association among the presence of metabolic syndrome (MetS), infection of hepatitis B virus and the clinicopathological parameters of patients with pancreatic cancer.

Variate	Hepatitis B Virus (HBV) Infection (Number of patients)			Metabolic syndrome (MetS) (Number of patients)		
	HBsAg-negative andAnti-HBc-negative(n = 266)	HBsAg-positive andAnti-HBc-positive(n = 64)	*P* value	No (n = 415)	Yes (n = 73)	*P* value
Age (median±SD)	**60.50±10.747**	**52.00±11.155**	**0.001**	**57.00±10.974**	**59.00±11.185**	**0.038**
Age (<65/≥65)	**169/97**	**55/9**	**0.001**	**295/120**	**43/30**	**0.038**
Gender (Male/Female)	**181/85**	**53/11**	**0.020**	287/128	45/28	0.204
Pretherapeutic weight (Normal orloss <5 per cent/loss ≥5 per cent)	94/172	23/41	0.928	158/257	30/43	0.624
Carcinoembryonic antigen 199(CA19–9) (Normal/Elevated)	56/202	19/41	0.102	106/287	12/57	0.092
Location of tumor (Head/Body/Tail/Diffuse)	171/41/33/21	45/11/4/4	0.509	276/65/44/30	45/9/12/7	0.395
**The 7^th^ tumor-node-metastasis (TNM) staging (AJCC) (Ia +** **Ib + IIa + IIb/III + IV)**	75/191	15/49	0.443	**136/279**	**7/66**	**0.000**
**Degree of differentiation** **(Well differentiated/Moderate differentiated/Poorly differentiated or mucinous adenocarcinoma/Not** **documented)**	26/63/99/78	4/16/29/15	0.522	**41/107/147/120**	**4/10/38/21**	**0.008**

## Discussion

To the best of our knowledge, this is the first study to determine the prognostic effects of several risk factors and survival in patients with pancreatic cancer. Although previous studies have provided evidence in support of the association between the ABO blood type, smoking, alcohol consumption, obesity, diabetes, HBV infection and increased risk of pancreatic cancer, our study did not find an effect of smoking, alcohol drinking, or the ABO blood group on the prognosis of patients with pancreatic cancer. However, patients with HBsAg-positivity and elevated fasting plasma glucose levels were associated with unfavorable survival; however, these were not independent prognostic factors. The presence of MetS was better than hyperglycemia, and MetS was independently associated with OS.

Recently, several epidemiological observations have found a relationship between ABO blood group genotypes and pancreatic cancer risk [17], [18], [19], [23]. Alterations in ABO-blood-group-related genotypes consists of the major tumor-related aberrant glycosylation, which may lead to the formation of cancer-related carbohydrate antigens [39]. Basic research has found that alterations in glycosyltransferase, which is specifically involved in the processes of modification of intercellular adhesion, cellular membrane signaling [40] and malignant-cell immunosurveillance [41], may also occur during tumorigenesis. Glycosyltransferase-related coding genes may also be considered as candidate prognostic factors. Two studies investigating the prognostic effects between the ABO blood type and pancreatic cancer have shown inconsistent results [36], [37]. Andrea Wang-Gillam et al. [37] demonstrated that non-O blood types did not affect OS among patients who underwent resection for pancreatic cancer. However, patients with locally advanced and metastatic disease were not specifically evaluated in their study. Qi-wen Ben et al. [36] found that, in patients who underwent a potentially curative resection, the median OS of patients with blood type O was significantly longer than those with non-O blood types. However, there was no significant difference in the OS of all stages of patients. In the present study, which included all patient stages, we could not find an association between the ABO blood type and cancer mortality.

Some epidemiological observations have found an association between HBV infection and the risk of pancreatic cancer development [20], [21], [22]. However, infection also can trigger regional inflammatory responses. Inflammatory processes always accompany cancer. The inflammatory microenvironment also plays decisive roles in tumor progression through the recruitment of various immunocytes and proinflammatory cytokines that influence patient prognosis [42]. Nevertheless, the prognostic effect of HBV infection in patients with pancreatic cancer has not been well examined. In the present study, we found that HBsAg-positivity was the most significant predictor of OS in a univariate analysis; however, this factor did not remain independent or significantly different in a multivariate analysis. We attempted to tease out the influence of other prognostic factors and HBV infection on the survival of patients with pancreatic cancer. Interestingly, we found that patients who were chronic carriers of HBV infection had an earlier onset of disease and were more predominantly male compared to those who were never infected. However, HBV infection was not associated with other significant clinicopathological prognostic factors. We considered that a genetic mutation or immune disorder mechanism may be involved in the development and progression of HBsAg-positive pancreatic cancer and may therefore contribute to an earlier disease onset, sex disparity and influence of prognosis.

Diabetes has been consistently linked to the risk of pancreatic cancer. However, studies investigating the prognostic value of diabetes mellitus and pancreatic cancer have shown inconsistent results [25], [26], [27], [28]. M. A. Shama et al. [26], Busaidy et al. [27] and Jee, S. H. et al. [28] demonstrated that diabetes may be an independent prognostic factor in patients with pancreatic cancer. Nevertheless, data from the Veterans Affairs Central Cancer Registry (VACCR) showed that patients with diabetes mellitus do not have worse OS [25]. Obesity is another risk factor for pancreatic cancer. Obesity is associated with a lower OS in patients with pancreatic cancer [29]. Adiposity in obese patients has been shown to be strongly associated with insulin resistance and disorders of lipid metabolism [31]. These several risk factors, which commonly appear together, have been defined as MetS. There are several pathogenetic factors promoting the progression of pancreatic cancer among patients with MetS. Several studies have shown that the altered secretion of adipokine production, the activation of insulin growth factor signal pathway [33], a pro-inflammatory state [34], [35], [43], a pro-coagulant state [44], and alterations of genetic factors [45] may promote the development and progression of cancer. A proinflammatory state is recognized by elevated infiltrating macrophages, T cells, tumor necrosis factor-alpha and C-reactive protein [34], [35], [43] in the tumor microenvironment, and it is commonly present in patients with MetS. Elevated plasminogen activator inhibitor-1 is mostly associated with MetS, creating a prothrombotic state. The activation of Akt and the mammalian target of the rapamycin signaling pathway are involved in mice with diet-induced obesity and are associated with the activation of the IGF-I and epidermal growth factor receptors [45]. MetS has been identified as an independent risk factor for pancreatic cancer in some population-based studies [9], [10], [11], [12]. However, the association between MetS and cancer-related survival in patients with pancreatic cancer has been limited thus far. In this study, we found that in patients with elevated fasting plasma glucose levels, elevated glucose was not an independent predictor for OS. However, the presence of MetS was a better predictor than other metabolism-related factors, and it was independently associated with reduced OS. MetS has also been shown to be linked with advanced tumor stage and reduced cancer-related survival in other cancers [46]. In the present study, we found that patients who presented with MetS had later disease staging and a lower degree of pathological differentiation than those without MetS.

Recently, several clinical studies have found a reduced incidence of tumors treated with the antidiabetic agent metformin [47], patients with hypertension treated with beta blockers [48], angiotensin receptor blockers (ARBs) losartan [49] and angiotensin-converting enzyme inhibitors [50], as well as potential antiproliferative and proapoptotic effects of these treatments. Based on our results, we suggest the potential survival benefit resulting from the modification of MetS in patients with pancreatic cancer as an interesting future research focus.

There are some limitations to this study. First, only 18 patients (3.69%) were categorized as obese in our study. Due to the lack of a direct measure of central adiposity in this study, BMI was utilized as a proxy variable for waist circumference [38], though BMI may be less informative. Few patients were categorized as obese may also be attributed to racial differences between Western and Eastern patients. Second, detailed information on the use of medications (e.g., metformin, ARBs losartan, and the beta blockers propranolol or atenolol) that may modify metabolic risk factors and cancer prognosis was not available. Third, the performance status of patients was not obtained. Finally, because of the lack of a definite time of tumor recurrence, the association between MetS and recurrence-free survival (RFS) had not been performed in patients who had undergone macroscopically radical surgery.

Overall, our study did not provide evidence of the impact of the ABO blood group, elevated fasting plasma glucose, obesity, smoking, alcohol consumption or HBV infection on prognosis in patients with pancreatic cancer. However, we report for the first time that patients with chronic HBV infection may represent a special subtype, who present with earlier disease onset and male dominancy. Patients with MetS had later disease staging and a poorer histologically differentiated grade than those without MetS. Patients with MetS demonstrated significantly poorer survival. Additional large-scale studies are needed to extend and confirm our results.
